# Vitamin D levels in healthy men in eastern Saudi Arabia

**DOI:** 10.4103/0256-4947.55168

**Published:** 2009

**Authors:** Mir Sadat-Ali, Abdulmohsen AlElq, Haifa Al-Turki, Fatma Al-Mulhim, Amein Al-Ali

**Affiliations:** a From the Department of Orthopedic Surgery, College of Medicine, King Faisal University, Al-Khobar, Saudi Arabia; b From the Department of Internal Medicine, King Fahd Hospital of the University, Al-Khobar, Saudi Arabia; c From the College of Medicine-Obstetrics and Gynecology, King Faisal University, Al-Khobar, Saudi Arabia; d From the College of Medicine-Radiology, King Faisal University, Al-Khobar, Saudi Arabia; e From the College of Medicine-Biochemistry, King Faisal University, Al-Khobar, Saudi Arabia

## Abstract

**BACKGROUND:**

Studies in 1980s and 1990s indicated that vitamin D levels in the ethnic Saudi Arabian population were low but no studies since that time have evaluated vitamin D levels among healthy young or middle-aged Saudi men. Thus, we assessed the serum level of 25-hydroxyvitamin D (25OHD) among healthy Saudi Arabian men living in the Eastern Province.

**SUBJECTS AND METHODS:**

One hundred males aged 25-35 years (the age range of peak bone mass) and 100 males aged 50 years or older were randomly selected and evaluated clinically, including measurement of serum calcium, parathyroid hormone (PTH) and serum 25OHD levels. Vitamin D deficiency was defined as a serum level of 25OHD of ≤20 ng/mL and insufficiency as a serum level between >20 ng/mL and <30 ng/mL and normal ≥30 ng/mL.

**RESULTS:**

The mean (SD) age of subjects in the younger age group was 28.2 (4.5) years. Twenty-eight (28%) had low 25OHD levels; 10 (10%) subjects were vitamin D deficient with a mean level of 16.6 (3.4) ng/mL and 18 (18%) were vitamin D insufficient with a mean level of 25.4 (2.7) ng/mL. In the older age group, the mean age was 59.4 (15.6) years and 37 (37%) had low 25OHD; 12 (12%) subjects were deficient with a mean 25OHD level of 16.7 (3.4) ng/mL and 25 (25%) were insufficient with a mean 25OHD level of 25.3 (3.3) ng/mL.

**CONCLUSION:**

The prevalence of vitamin D deficiency among healthy Saudi men is between 28% to 37%. Vitamin D deficiency among young and middle age Saudi Arabian males could lead to serious health consequences if the issue is not urgently addressed.

The two main forms of vitamin D are vitamin D2 or ergocalciferol, which is obtained from plant and foods such as mushrooms, fish and egg yolk, and vitamin D_3_ or cholecalciferol, which is formed in the skin after exposure to sunlight or ultraviolet light. Vitamin D is important for calcium homeostasis and essential for skeletal health. Deficiency of vitamin D is responsible for the development of rickets in children and osteomalacia in adults.^1^ There is no absolute consensus on the cut-off value between a normal and low level of vitamin D. Recently, many studies have used 32 ng/mL as a cut-off value and most experts now recommend the normal level of 25-hydroxyvitamin D (25OHD) to be ≥30 ng/mL. They have also agreed to define vitamin D insufficiency as a level of >20-29 ng/mL and deficiency when the level is ≤20 ng/mL.^2^ There are many causes of vitamin D deficiency, which are related to decreased synthesis, deceased bioavailability, increased catabolism or demands and increased urinary loss. It has been estimated that around one billion persons worldwide have vitamin D deficiency or insufficiency.^3^ Saudi Arabia is one of the sunniest areas of the world and exposure to sunlight might be assumed to be sufficient to maintain adequate vitamin D status. However, vitamin D deficiency is common among the Saudi population. As early as 1982 Woodhouse and Norton^4^ reported low vitamin D levels in the ethnic Saudi Arabian population. Later, Sedrani and colleagues confirmed the earlier finding,^5^ and also found that low vitamin D levels were not related to one region, sex, age or season.^6^ Despite these reports no action was taken to adequately fortify foods or encourage people to increase their vitamin D intake. Recent attention to the high prevalence of osteoporosis and its association with low vitamin D levels in adults has raised the importance of vitamin D evaluation. In addition to many other risk factors, a low level of vitamin D is considered to be one of the most important risk factors for osteoporosis and related fractures.^7^,^8^ Chapuy^9^ reported that adequate vitamin D intake can prevent hip fractures while Lips et al^10^ have shown that vitamin D is essential for calcium metabolism as well as for fracture prevention. In the last 3 to 4 decades the dietary habits and lifestyle of children and adults in Saudi Arabia has changed tremendously,^11^ but vitamin D levels among Saudis have not been reassessed. To our knowledge, no recent studies have evaluated vitamin D levels among healthy young or middle-aged Saudi men. This objective of this study was assessment of serum 25OHD levels among healthy Saudi males.

## SUBJECTS AND METHODS

This cross-sectional study was carried out over a 3-month period between 1 February 2008 and 31 May 2008 at King Fahd Hospital of the University-Al Khobar, located in the Eastern Province of Saudi Arabia. The study was approved by the research and the ethical committee of the Medical College at King Faisal University, Dammam. Two hundred healthy Saudi Arabian men were randomly selected, including 100 males aged 25 to 35 years (the age range of peak bone mass) and 100 males aged 50 years or older. Exclusion criteria were the presence of chronic diseases that affect vitamin D status such as malabsorption, chronic liver disease, renal impairment or nephritic syndrome, the use of vitamin D supplements, or drugs that can affect vitamin D metabolism such as anticonvulsants or corticosteroids, and a family history of hypocalcemia or vitamin D disorders. Informed verbal consent was obtained from all the candidates. Demographic data such age and sex in addition to information on sunlight exposure for the purpose of obtaining vitamin D and the frequency and amount of dairy product consumption were obtained through a questionnaire. Subjects 25 to 35 years of age were recruited from medical students, interns, and residents in addition to hospital employees. Men aged 50 years and older were randomly selected from visitors to the hospital and examined clinically to rule out the presence of any disease of exclusion. Blood samples were taken from all subjects in the morning and after an overnight fast. Samples were kept frozen at −20°C until the time of analysis. Serum calcium, serum phosphorous, alkaline phosphatase and parathyroid hormone levels were determined by standard laboratory procedures. Serum levels of 25-hydroxyvitamin D3 (25OHD3) were measured by radioimmunoassay using the Wallac 1470 Gamma Counter (Wallac Inc, Gaithersburg, MD, USA). 25OHD3 were considered normal if >30 ng/mL. Vitamin D deficiency was defined as a serum level of 25OHD of ≤20 ng/mL and insufficiency as a serum level between >20 ng/mL and < 30 ng/mL. The data were analyzed using SPSS (Statistical Package for the Social Sciences), version 14.0, Chicago, Illinois. Data were expressed as the mean and standard deviation. Statistically significant differences between groups were determined with the t test. *P* values of less than .05 (*P*<.05) using a confidence interval (CI) of 95% were considered significant.

## RESULTS

In the men aged 25 to 35 years, 72 (72%) had a normal level of 25OHD3 and 28 (28%) had a low level, of whom 18 (18%) were insufficient and 10 (10%) were deficient (Table 1). The mean age of subjects in the three groups were similar. In the men aged 25 to 35 years, the mean levels of the 25OHD3 were significantly lower in the subjects who were insufficient and deficient as compared to the normal subjects. When compared to normal subjects, serum calcium was significantly lower in insufficient subjects (*P*=.002) whereas alkaline phosphatase and parathyroid hormone levels were higher in deficient subjects, though they were still within the normal range. Fifteen patients in the three groups consumed dairy products equivalent to about 250 mL of milk per day and there was no statistically significant difference between the groups. Only 6 patients reported exposure to sunlight for the sake of obtaining vitamin D. For subjects aged 50 years and older, sixty-three (63%) had normal 25OHD3 levels while levels were low in 37 subjects (37%) (Table 2). Of those with low 25OHD3 levels, 25 (25%) were insufficient and 12 (12%) were deficient. Mean levels of 25OHD3 were significantly different between the three groups. Deficient subjects were older than normal subjects (*P*=.001). Alkaline phosphatase was significantly higher in insufficient and deficient subjects and parathyroid hormone levels, though in the normal range, were higher in insufficient and deficient subjects when compared to normal subjects (*P*=.002). Few patients in this age group consumed dietary products or exposed themselves to sunlight.

**Table 1:** Clinical and demographic data for healthy men aged 25-35 years (n=100).

	Normal 25OHD (≥30 ng/mL) (A)	25OHD insufficiency (>20-<30ng/mL) (B)	250HD deficient (≤20 ng/mL) (C)	*P* between A and B	*P* between A and C
Number of subjects	72	18	10		
Mean (SD) age (years)	27.9 (3.5)	28.9 (4.3)	28.5 (4.5)	.1	.1
Exposure to sunlight (min/day)	5	0	1	.01	.01
Adequate consumption of dairy products (250 mL/day)	9	2	4	.8	.02
Calcium level (mg/dL) (normal, 8.5-10.5)	9.5 (0.6)	9.1 (0.3)	9.2 (0.5)	.002	.3
Phosphorus (mg/dL) normal, 2.5-4.9)	3.9 (0.6)	3.6 (0.4)	3.7 (0.7)	.1	.1
Alkaline phosphatase (IU/L) (normal, 50-140)	87.6 (31.7)	85.9 (17.9)	105.5 (16.2)	.2	.01
Parathyroid hormone (pmol/L) (normal, 1.3-7.6)	5.0 (2.2)	5.2 (1.5)	6.9 (2.7)	.1	.06
25-hydroxyvitamin D3 (ng/mL) (normal, >30 ng/mL)	43.8 (6.4)	25.4 (2.7)	16.6 (3.4)	.01	.01

Values are mean (standard deviation) unless noted otherwise.

**Table 2:** Clinical and demographic data for healthy men aged 50 years or older (n=100).

	Normal 25OHD (≥30 ng/mL) (A)	25OHD insufficiency (>20-<30ng/mL) (B)	250HD deficient (≤20 ng/mL) (C)	*P* between A and B	*P* between A and C
Number of subjects	63	25	12		
Mean (SD) age (years)	58.7 (8.1)	58.3 (9.7)	64.8 (15.7)	.2	.001
Exposure to sunlight (min/day)	9	2	3	.1	.4
Adequate consumption of dairy products (250 mL/day)	12	3	2	.8	.4
Calcium level (mg/dL) (normal, 8.5-10.5)	9.0 (0.7)	9.2 (0.3)	9.1 (0.2)	.9	.3
Phosphorus (mg/dL) (normal, 2.5-4.9)	3.8 (0.5)	3.9 (0.6)	3.5 (0.2)	.2	.3
Alkaline phosphatase (IU/L) normal, 50-140)	87.1 (32.4)	84.8 (26.3)	109.5 (19.2)	.1	.001
Parathyroid hormone (pmol/L) (normal, 1.3-7.6)	5.2 (3.4)	5.7 (0.6)	6.4 (0.7)	.1	.002
25-hydroxyvitamin D3 (ng/mL) normal, >30 ng/mL)	39.8 (5.9)	25.3 (3.2)	16.7 (3.4)	.001	.001

Values are mean (standard deviation) unless noted otherwise.

## DISCUSSION

There are many risk factors for vitamin D deficiency.^3^ Rickets, which is predominately related to vitamin D deficiency, was highly prevalent during the early twentieth century when more than 80% of children living in industrialized cities in Europe and North America suffered from the disease,^3^ After the initiation of food-fortifying programs, it was assumed that vitamin D deficiency was eradicated and no longer a health issue in Western and developed countries.^12^ However, recent reports indicate that vitamin D deficiency is still rampant, especially among the elderly and it continues to be an unrecognized epidemic in many populations worldwide.^12^ Dharmarajan et al^13^ reported that 54% of their patients had suboptimal vitamin D levels. In general, the prevalence of vitamin D deficiency (<20 ng/mL or 50 nmol/L) in otherwise healthy young adults 18 to 29 years is 36% and it is much higher among black women, elderly people and general medicine inpatients.^14^ Vitamin D deficiency has many health consequences. It has long been recognized that both insufficiency and deficiency of vitamin D can cause serious effects on the skeleton and other systems.^12^,^15^

This study from the eastern region of Saudi Arabia raises a concern of a health issue for the Saudi population at large. Twenty-eight percent of healthy men at the age of peak bone mass (25 to 35 years of age) and 37% of healthy men aged 50 years and older in this cross-sectional study were suffering from low vitamin D levels (insufficiency and deficiency). This prevalence is higher than the previously reported figure of 20% from the Eastern Province.^3^ Also, this prevalence is much higher than what was reported by Atli et al,^16^ who found vitamin D deficiency in only 15.3% of elderly males from Turkey. On the other hand, the prevalence of vitamin D deficiency among our subjects was lower than that reported recently by O'Sullivan et al^17^ who found low 25OHD levels in 54% of their healthy young men with an average age of 36 years. Very low vitamin D levels have been reported from other Middle Eastern countries, particularly from Lebanon, Iran, Jordan and Turkey.^18^–^20^

Of great concern is the finding that 28% of healthy men aged 25 to 35 years had low 25OHD. Vitamin D is essential for the attainment of adequate peak bone mass and it plays a key role in bone development and bone density. Not reaching the expected peak bone mass at this age may predispose subjects to the development of osteopenia and osteoporosis at a later age.^21^ This can partially explain our previous finding of a high prevalence of osteopenia and osteoporosis among Saudi men older than 50 years of age.^22^ In the current study, a low vitamin D level was found to be prevalent among our healthy subjects older than 50 years and as expected, the mean age of patients with deficiency was older than that of patients with a normal vitamin D level. This finding should alert us to the importance of proper and adequate supplementation of vitamin D when treating patients with osteopenia or osteoporosis.^10^ Despite the presence of low vitamin D among subjects in both age groups, serum calcium and parathyroid hormone were in the normal range, which supports the importance of assessment of vitamin D levels if clinically indicated.^23^ Although our subjects were healthy and active and despite the fact that Saudi Arabia is one of the sunniest countries, with sun available most days throughout the year, few patients in either age group had sensible sunlight exposure for the sake of obtaining vitamin D, which is considered the main source of vitamin D.^24^ Avoidance of sunlight might be a contributing factor to the high prevalence of low vitamin D in our population. The cultural practice of wearing long sleeves and head covers that also cover part of the face is unlikely to be the major cause of vitamin D deficiency among our subjects. In fact Sedrani et al^5^ found adequate serum 25OHD levels among Saudi female students who were veiled and more extensively clothed. The finding of Sedrani et al^5^ stresses the importance of public education about the importance of making time for sun exposure for the sake of obtaining vitamin D. On the other hand, only 15% of the younger group and 17% of the older group consumed an adequate amount of dairy products, which again indicates a lack of public awareness about the importance of adequate calcium intake.

The time of year is an important factor in measurement of vitamin D levels in the diagnosis of insufficiency or deficiency. Bolland et al^25^ found that summer is the ideal time to measure vitamin D levels as there is seasonal variation with a 14% increase 25(OH)D concentrations in men in the summer,^26^ but Saadi et al^27^ found that mean serum 25OHD was highest in April, which marks the end of the winter season. We measured the 25OHD in the spring/summer season, which could have been the highest level of 25OHD3.

Our study has a few limitations. The study sample of 200 healthy individuals was small and the fact that blood samples were collected only once during the months of February to May. It would be useful if patients were evaluated at different times of the year to study the seasonal variation. Also the Eastern Province of Saudi Arabia is located on the coast of the Arabian Gulf and high fish consumption would be expected, but unfortunately, this information was not collected from the subjects.

In conclusion, even though not representative of all Saudi men living in the country, our study suggests that low vitamin D is prevalent in apparently healthy Saudi men. The deficiency of vitamin D might be related to lack of adequate exposure to sunlight and possibly inadequate consumption of dairy products. We believe that there is an urgent need for public education about the role of vitamin D in health to avoid the complications of vitamin D insufficiency and deficiency.
